# Roll Eccentricity Detection and Application Based on SFT and Regional DFT

**DOI:** 10.3390/s23167157

**Published:** 2023-08-14

**Authors:** Kexin Yang, Gang Zheng, Zhe Yang

**Affiliations:** 1 Faculty of Automation and Information Engineering, Xi’an University of Technology, Xi’an 710048, China; zhenggang@xaut.edu.cn (G.Z.); cice@xaut.edu.cn (Z.Y.); 2 Shaanxi Key Laboratory of Complex System Control and Intelligent Information Processing, Xi’an University of Technology, Xi’an 710048, China

**Keywords:** eccentric disturbance compensation, SFT, regional DFT

## Abstract

Roll eccentricity disturbance is a high-frequency periodic change signal caused by the irregular shape of the roll and roll bearing, which is difficult to identify and affects the periodic deviation of the exit thickness of the strip. To achieve rapid identification of the source and a mathematical model of roll eccentricity signals, a sparse Fourier transform (SFT) and regional DFT method for roll eccentricity signal recognition and detection was proposed. This method utilizes SFT to calculate the signal FFT more quickly based on the sparsity of the signal frequency domain. Under the premise of knowing the roll diameter, the signal frequency spectrum is identified online, the amplitude and phase are identified through regional DFT, and the eccentricity disturbance is compensated on site. The simulation results show that this method can accurately identify the source of roll disturbance, quickly update and replace the problematic rolls, and improve the online recognition efficiency by more than 3000 times. This method has good results in online detection and recognition of roll eccentricity signals, greatly improving engineering application efficiency, and ultimately achieving the goal of improving the accuracy of strip outlet thickness.

## 1. Introduction

The irregular shape of the rollers and roller bearings during the rolling process of plate and strip steel can cause non-circularity of the rollers themselves, as well as separation between the geometric axis of the roller body and the rotating axis, resulting in eccentric disturbance of the rollers. The eccentric disturbance of the rolling mill roll can cause high-frequency periodic changes in the roll gap during actual operation, resulting in a certain periodic deviation in the outlet thickness of the strip. The thickness of the strip outlet is an important indicator for measuring the quality of the plate and strip steel [1], and its value plays a decisive role in the quality of the steel plate. In the actual production line of strip, it is necessary to quickly and accurately analyze the information on roll eccentricity disturbance and reverse-compensate it into the AGC control system, in order to solve the interference problem of eccentricity disturbance more efficiently and accurately and achieve the goal of improving the accuracy of strip outlet thickness.

This article introduces the formation and mathematical model of roll eccentricity disturbance, and uses SFT to calculate signal FFT more quickly based on the sparsity of signal frequency domain. Under the premise of knowing the diameter of the rolling roller, in order to improve operational efficiency and achieve online identification of eccentric disturbance data, a regional DFT method is proposed to identify the signal spectrum online and identify the amplitude and phase, compensate for eccentric disturbance on site, and ultimately achieve the goal of improving the accuracy of strip outlet thickness.

## 2. Introduction to Roll Eccentricity Disturbance

**Figure 1:**
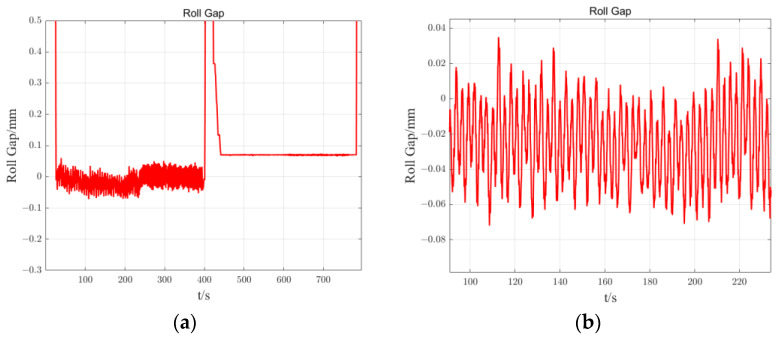
Actual roll gap with eccentric disturbance: (**a**) the actual roll gap in the first 400 s; (**b**) amplification of the portion of (**a**) that contains eccentric disturbances.

The eccentricity disturbance of the roller is caused by the irregularity of the shape of the roller and the roller bearing, which mainly occurs during manufacturing, grinding, thermal expansion, and other processes, resulting in the non-circularity of the roller itself and the separation of the geometric axis of the roller body from the rotational axis. The eccentric disturbance of the rolling rolls causes high-frequency periodic changes in the actual gap of the rolling mill, resulting in periodic deviations in the thickness of the strip outlet [1]. The eccentricity disturbance of the rolling roller causes a deviation in the thickness of the strip outlet, while also reducing the adjustment quality of the conventional regulator, affecting the thickness value of the strip and causing fluctuations. The actual roll gap in the first 400 s of Figure 1a experienced high-frequency periodic changes due to the eccentricity of the rolls. By amplifying the part containing the eccentricity disturbance in Figure 1a to obtain Figure 1b, it can be seen that the actual roll gap contains periodic eccentricity disturbance, which in turn causes deviations in the thickness values of the strip produced subsequently.

The eccentricity disturbance of the rolling mill has the following three characteristics [2]:

1. Periodicity: In signals such as rolling force, roll gap, tension, or thickness, there may be roller eccentricity disturbance signals, which can be seen as a series of sine periodic waves with a frequency proportional to the roller speed.
2. Variability: Due to the variable speed of the roller, the frequency of eccentric disturbance also varies. When the rolling speed changes, the frequency of the eccentric disturbance signal also changes accordingly.
3. Complexity: There are various random noises generated in the eccentric disturbance signal, such as collection noise, changes in hardness and thickness of the rolled piece, and changes in oil film thickness. The analysis of the disturbance signal is complex.

### 2.1. Mathematical Model of Roll Eccentricity

The eccentricity disturbance of the rolling mill can be seen as a series of sinusoidal periodic signals with a frequency proportional to the rolling mill speed [3]. The fundamental and second harmonic components generally play a major role. According to the characteristics of eccentric disturbance, the interference signal that causes fluctuations in outlet thickness includes complex periodic signals with multiple periodic components, including periodic signals with different frequencies such as upper and lower support roller eccentricity, upper and lower work roller eccentricity, upper and lower intermediate roller eccentricity, and inlet thickness fluctuation. Assuming that the eccentric disturbance signal contains periodic components of n frequencies, the eccentric disturbance has the following mathematical form:

(1) $$A_{1}\cos\left(w_{1}+\theta_{1}\right)+A_{2}\cos\left(w_{2}+\theta_{2}\right)+\ldots +A_{n}\cos\left(w_{n}+\theta_{n}\right).$$

If the parameters such as frequency, amplitude, and phase angle of each signal can be identified, and the signal is added to the compensation end of the AGC system in reverse, the impact of roll eccentricity disturbance can be eliminated, and the accuracy of strip outlet thickness can be improved.

### 2.2. Roll Eccentricity Disturbance Compensation Control Method

The compensation control for roll eccentricity disturbance can be divided into three methods: passive control, active compensation method, and preventive control method. The main representative of the passive control method is the dead zone method, which only filters out the eccentric disturbance signal in the signal and does not compensate for the signal. The main function of this method is to make the AGC system no longer sensitive to the thickness interference caused by the eccentric disturbance system, and it essentially cannot reduce or eliminate the impact of eccentric disturbance. The active compensation method is currently the most studied method, and extracts the roll eccentricity signal from some rolling signals that reflect the roll eccentricity through various mathematical calculation methods, and reversely compensates the roll eccentricity disturbance signal to the control system of the rolling mill. The active compensation method is mainly divided into the analytical and the comprehensive method. Preventive control is mainly aimed at the process aspect, that is, making corrections on the rolling mill to improve the surface-processing accuracy of the rolls and bearings, improve installation accuracy, and eliminate the impact of roll eccentricity.

With the development of computer science control and signal processing mathematical methods, new compensation control methods have emerged, such as the dead zone method, Newman method, Smith method, constant-rolling-force closed-loop control method, adaptive filtering method, Fourier transform method, wavelet analysis method, neural network method, etc. The dead zone method is a typical example of passive eccentricity control methods. The dead zone method can remove periodic signals from the control signal, so that the adjustment signal caused by an eccentric disturbance signal does not enter the control system. The disadvantages of this method are low accuracy, that the width of the dead zone conflicts with the control accuracy, that the dead zone is too large, that the control accuracy is low, that the dead zone is small, and that the suppression effect on eccentric disturbances is too poor. The Newman method involves installing a cam to rotate together with the rotating roller. The displacement sensor is used to measure the eccentricity disturbance of the installed cam, which is transmitted through an electronic signal to the roller gap control adjuster. This method is difficult to operate and the compensation effect is average. Due to the main condition in the Smith method—the rectifier is made of metal material which generates noise signals during use with similar phase amplitude characteristics to the control signal—the method has poor ability to suppress second harmonic. The constant-rolling-force closed-loop control method is a typical representative of active compensation methods, and keeps the rolling force constant and eliminates the impact of eccentric disturbance when the rolled piece enters the roll gap. When the thickness of the incoming material changes, this method cannot automatically adjust the size of the roll gap. The adaptive filtering method analyzes the rolling force signal and extracts the eccentric disturbance signal [4]. The adaptive filter can use many adaptive algorithms, such as recursive least squares (RLS), least mean squares (LMS), etc. [5]. The Fourier transform method first extracts rolling feature signals containing eccentric disturbances, then performs Fourier spectrum analysis on the features and estimates the phase amplitude characteristic parameters of eccentric disturbances [6]. The computational complexity of Fourier transform is significant and cannot be widely applied to practical industrial sites. In order to improve the computational speed of Fourier transform, fast Fourier transform (FFT) has been developed, which expands the application range of Fourier transform from theory to industrial sites. The wavelet analysis method localizes the time-domain and frequency-domain decomposition of signals under different conditions, dividing signals of multiple frequencies, and filtering eccentric signals. Usually, in research, the rolling process is assumed to be a linear model, while the actual rolling process is a complex nonlinear process [7]. Especially in today’s increasingly high requirements for strip thickness accuracy, simplifying the model structure can lead to increased calculation errors. In order to improve accuracy, people began to study the application of neural networks in the field of roller eccentricity recognition. Firstly, various rolling feature parameters, such as rolling force and outlet plate thickness, were collected to establish a neural network system, namely, the roller eccentricity model [8].

## 3. Analysis of Roll Eccentricity Disturbance Data

Usually, the eccentricity signal of the roll is analyzed and extracted from data such as roll gap, thickness, or rolling force, and the mathematical model for obtaining the eccentricity of the roll is identified. Based on this mathematical model, the eccentricity signal is specifically calculated and compensated in reverse through the hydraulic servo system of the rolling mill [1]. This chapter mainly explores and studies how to identify and obtain eccentric disturbance models. By using methods such as SFT and regional DFT with known signal frequencies, the eccentricity disturbance signal is extracted from the rolling signal containing eccentricity disturbance.

### 3.1. Sparse Fourier Transform SFT

Discrete Fourier transform (DFT) is one of the most fundamental theoretical algorithms in signal processing [9]. The computational complexity of the fast algorithm FFT for DFT is O(nlogn). Compared with DFT, FFT greatly simplifies the computational complexity, but with the emergence of big data, FFT cannot fully meet the speed requirements. Therefore, it is necessary to further improve the calculation speed of FFT. In research, it has been found that many signals are sparse in the frequency domain [10], with only a small portion having large value points, while the values of most other points tend to approach 0. In January 2012, four researchers at the Massachusetts Institute of Technology, Dina Katabi, Haitham Hassanieh, Piotr Indyk, and Eric Price, proposed a faster method for obtaining FFT—sparse Fourier transform (SFT). Selective filtering of a portion of the input signal before the transformation begins greatly improves the computational speed. The computational complexity of SFT is $O(\log n\sqrt{nk\log n})$, where n is the signal length and k is the sparsity [10,11].

A signal x with a length of n and a spectrum X with only k non-zero coefficients, and other coefficients of 0 or approximately 0, is called a sparse signal or approximately sparse signal. If signal X satisfies the $l_{\infty }/l_{2}$ criterion, the signal can be well estimated. The core idea of SFT is to project signal frequency points into a set of “baskets” (with a number of B) according to certain rules [12]. Due to the sparsity of the signal, the frequency points of each maximum value will be projected into each “basket” with a high probability. The N-point sequence will be transformed into a B-point sequence, which will be used as the FFT of B-point. The signal reconstruction algorithm will be designed according to the basket-splitting rules to restore the spectrum of the original signal x. Nowadays, the main reconstruction algorithms include the aliasing congruence method, hash mapping method, phase decoding method, and binary search method. In this article, the hash mapping method is used.

- Random rearrangement of the spectrum:

Spectrum rearrangement is based on the displacement and scaling properties of DFT, as shown in Equation (2):

(2) $$p\left(n\right)=x\left(\sigma \left(n-a\right)\right)W_{N}^{\sigma bn},\;n\;\in \;\left[0,\;N-1\right].$$

Convert to frequency domain:

(3) $$P\left(\sigma (k-b)\right)=X(k)W_{N}^{\sigma bn},\;k\;\in \;\left[0,\;N-1\right].$$

where σ and N are mutually prime, and a and b are any integers. After the transformation of the k-point in the original spectrum to the position of σ(k−b)modN according to the complete residue theorem, when N and A are coprime, σ(k−b)modN is complete. To realize frequency domain rearrangement through time-domain transformation, take the form of $p\left(n\right)=x\left(\sigma n+\tau \right)$.

- Window function filter:

In SFT, only a part of the data is operated, and direct interception of the signal will lead to serious spectrum leakage. Therefore, the window function needs to be used to reduce the impact of spectrum leakage. The flat window function is characterized by a time domain and average energy concentration. The flat window function is designed by using the function and Gaussian window function. Order:

(4) h(n)=(sin⁡(2πf_c n))/πn,n∈ [−∞,∞]

$f_{c}$ is the cut-off frequency of the window function, and $f_{s}$ is the sampling frequency. If the passband frequency is set to N/B, then:

(5) $$f_{c}=\frac{1}{2B}f_{s}$$

Truncate point *h*(*n*) at point M=O(Blog⁡N) and shift point *M*/2 to the right.

Generate Gaussian window function with standard deviation A:

(6) $$g\left(n\right)=e^{-\frac{1}{2}(\frac{n}{\sigma }{)}^{2}},-\frac{M}{2}\leq n<\frac{M}{2}.$$

Fill in zero to point N. Then, the flat window function is:

(7) $$f\left(n\right)=h\left(n\right)g\left(n\right).$$

Filter the signal:

(8) q(n)=p(n)f(n).

The flat window function is introduced to make the passband flat and the stopband exponentially attenuated.

- Frequency domain down-sampling:

Down-sample the frequency domain *P*(*k*) at an interval of *N*/*B*, and divide the parameter B by N. Namely:

(9) Y(k)=P(kN/B), k∈ [0, B−1].

Down-sampling can be accomplished in the frequency domain by aliasing signals in the time domain.

(10) $$y(n)=\sum_{j=0}^{N/B-1}p(n+Bj),\;k\in \;[0,\;B-1].$$

Then perform an FFT operation on point B on the mixed signals to obtain *Y*(*k*).

- Signal reconstruction:

The FFT result *Y*(*k*) of point B was obtained, and the spectrum of the original signal was reconstructed using the hash mapping method. The hash function is defined as $h_{\sigma }(k)=round\left(\left(\sigma k{)}_{N}B/N\right)\right)$. Define the offset as $o_{\sigma }(k)=\sigma k-h_{\sigma }(k)(N/B)$.

Positioning cycle: the coordinates of *σk* maximum values in *Y*(*k*) are included in the set *J*, and the original image $I_{r}=\{k\in \;[0,\;N-1]|h_{\sigma }(k)\in J\}$, projected to *J*, is reflected through hashing.

Valuation cycle: each $k\in I_{r}$, $X_{r}(k)=Z(h_{\sigma }(k))W_{N}^{\tau k}/G(o_{\sigma }(k))$.

Each positioning cycle will result in a coordinate set $I_{r}$. In cycle $I_{r}$, if any coordinate $k\in I=I_{1}\cup I_{2}\cup \ldots \cup I_{l}$ occurs more than L/2 times, it is recorded in set $I^{\prime }$, and it is considered that set $I^{\prime }$ contains all target frequency points.

For each $k\in I^{\prime }$, take the median of L cycles as the final frequency value. This method reconstructs the frequency value of the original signal with a probability of (1−1/N).

### 3.2. Regional DFT

In industrial sites, the frequency of roll eccentricity disturbance is known, and it is necessary to calculate the amplitude and phase of roll eccentricity disturbance in real time online. Therefore, the method of calculating the amplitude and phase needs to be computationally small, and the accuracy within an acceptable range. The calculation result error should be within the following range:

Based on the above requirements, improve the DFT algorithm to achieve fast computation. The DFT algorithm calculates the frequency values of N points, but in practical applications, the frequency of the eccentric disturbance signal can be calculated based on speed. Therefore, only a small part of the value containing this frequency needs to be calculated. If the frequency of the eccentric disturbance signal is $f^{\prime }$, the DFT region range that needs to be calculated is defined as $\left[F_{L},\;F_{H}\right]$, $F_{L}<f^{\prime }<F_{H}$.

Input signal *X* (*N*), DFT calculation formula is:

(11) $$X(k)=\frac{1}{N}\sum_{n=0}^{N-1}x(n)e^{-j2\pi nk/N}$$

If the frequency domain accuracy of DFT is ds and the sampling frequency is Fs, then the number of data involved in the calculation is N=Fs/ds. According to Equation (11), calculate the values of $k=F_{L}/ds$ to $k=F_{H}/ds$.

## 4. Simulation Experiments and Data Analysis

**Figure 2:**
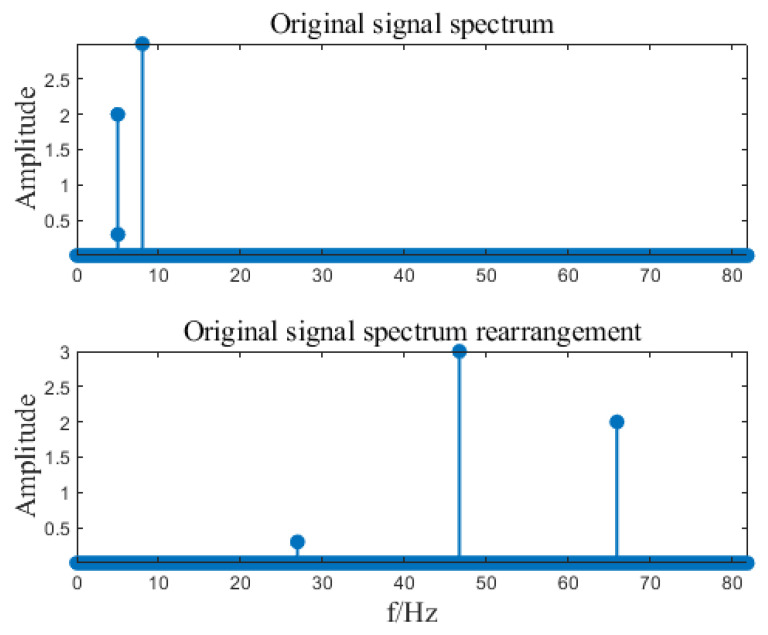
Spectrum rearranged randomly.

**Figure 3:**
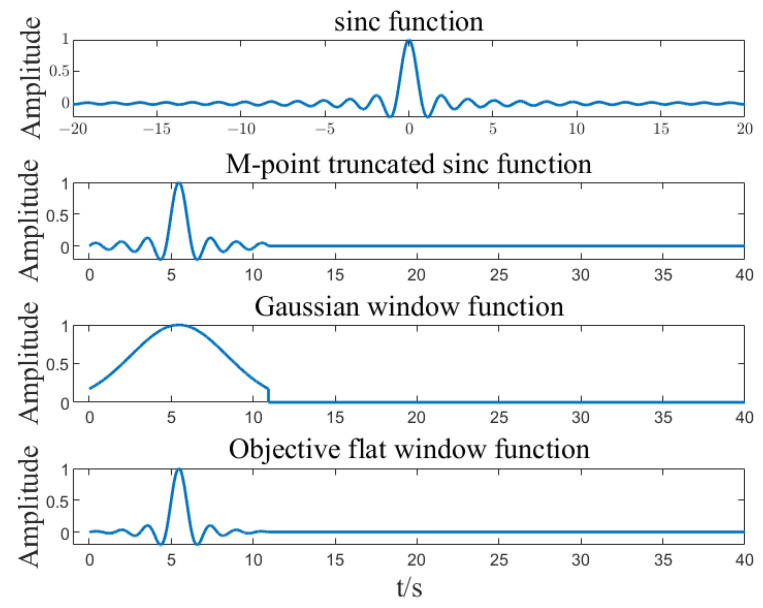
Flat filtering windows function.

**Figure 4:**
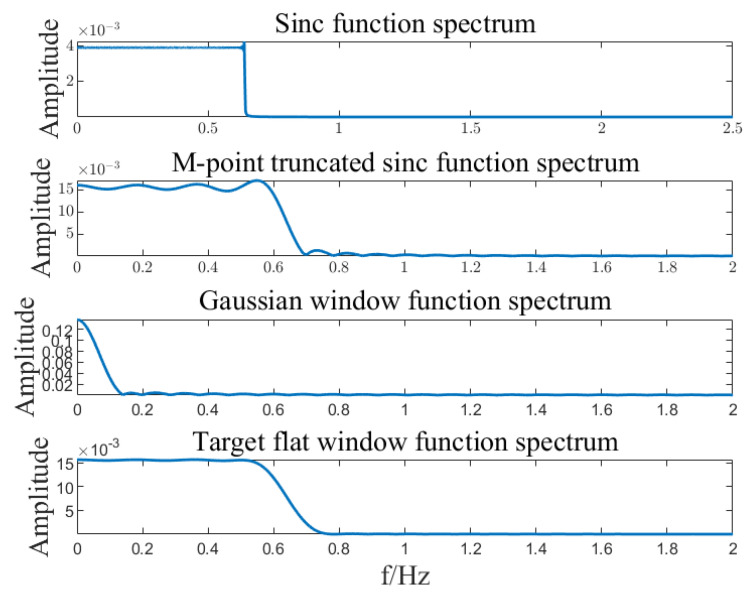
Flat filtering windows function r spectrum.

**Figure 5:**
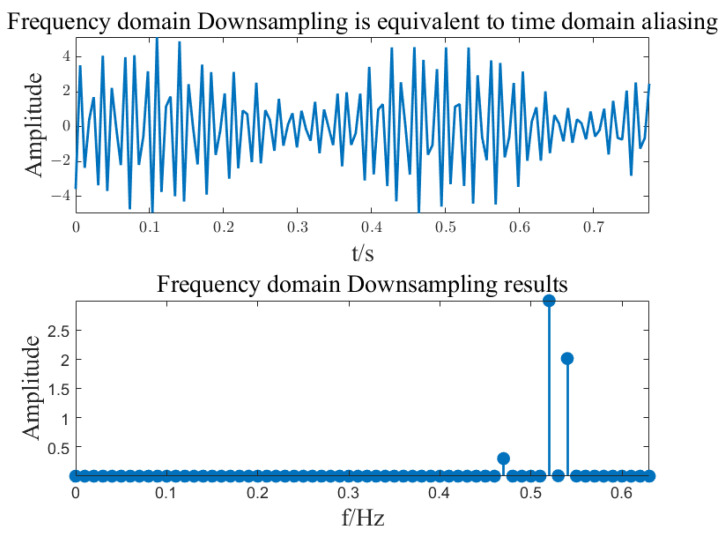
Subsampling of spectrum.

**Figure 6:**
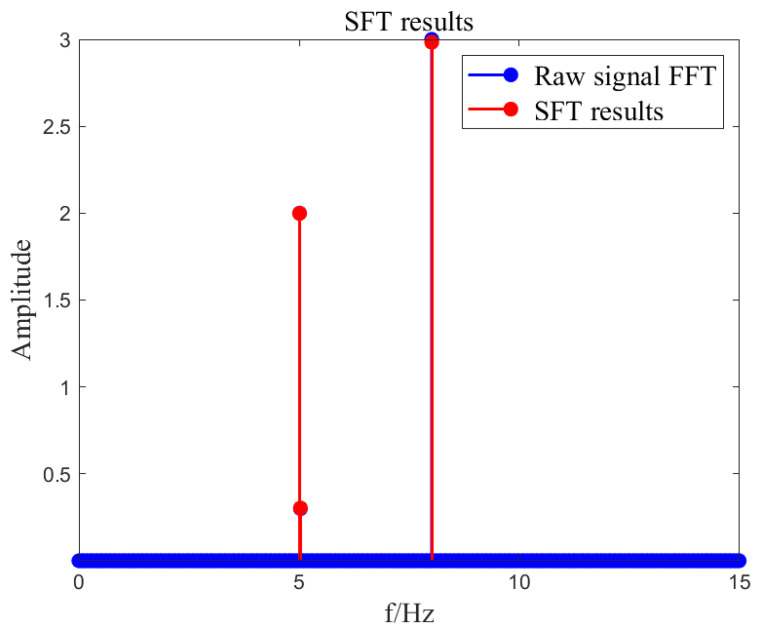
Signal reconstruction.

Input signal $x=A_{1}\cos(2\pi f_{1}t)+A_{2}\cos(2\pi f_{2}t)+A_{3}\cos(2\pi f_{3}t)$, where $A_{1}=2$, $A_{2}=3$, $A_{3}=0.3$, $f_{1}=5.01$, $f_{3}=8.01$, k=6, $B=2^{7}$. Calculate the SFT result of the input signal based on the SFT algorithm. The algorithm implementation is shown in Figure 2, Figure 3, Figure 4, Figure 5 and Figure 6. SFT extracts three signals at frequencies of 5.01 Hz, 5.03 Hz, and 8.01 Hz, and the signal amplitude calculation results are shown in Table 1.

**Figure 7:**
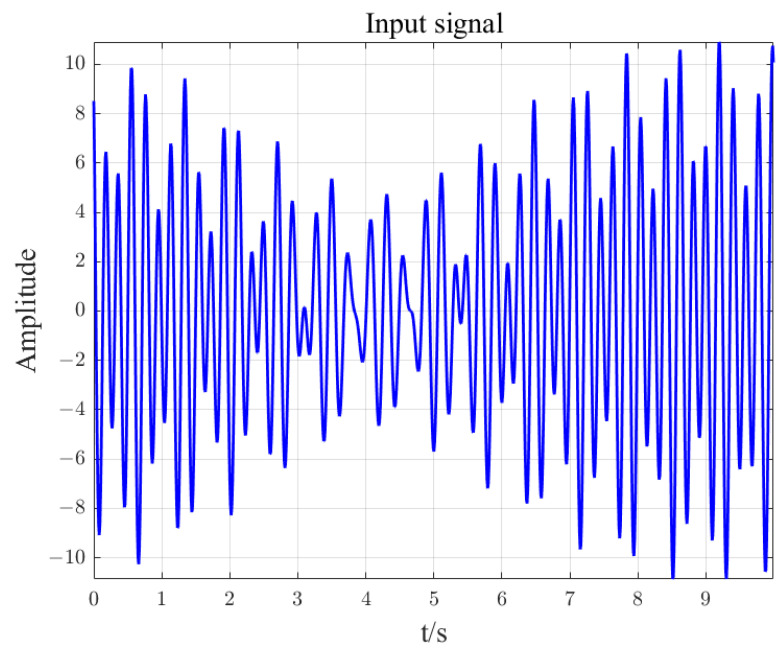
Input signal.

**Figure 8:**
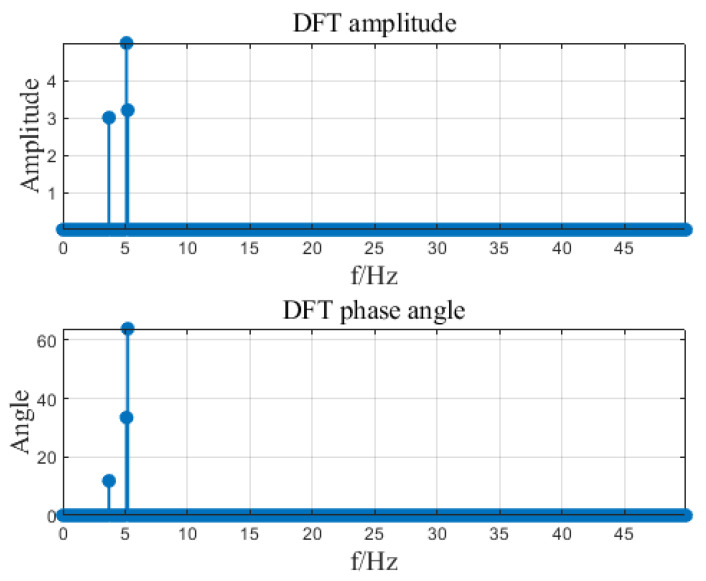
DFT calculation results.

**Figure 9:**
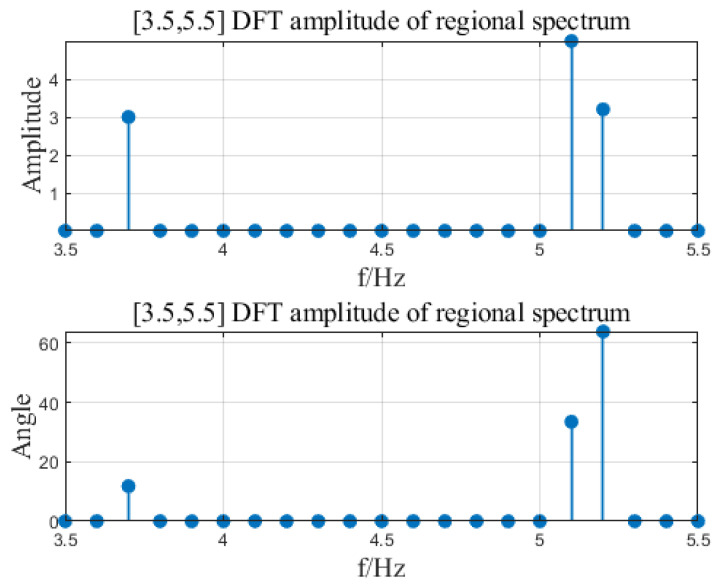
[$F_{L}$ = 3.5, $F_{H}$ = 5.5] area DFT calculation result.

**Figure 10:**
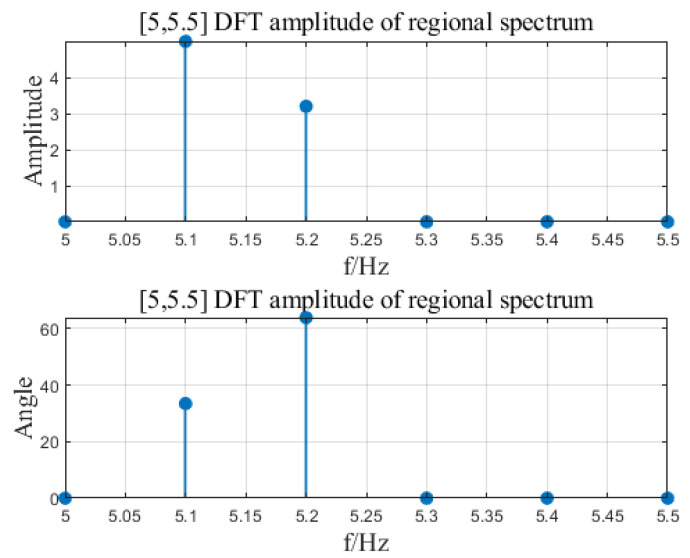
[$F_{L}$ = 5, $F_{H}$ = 5.5] area DFT calculation result.

Input signal $x=A_{1}\cos(2\pi f_{1}t+\theta_{1})+A_{2}\cos(2\pi f_{2}t+\theta_{2})+A_{3}\cos(2\pi f_{3}t+\theta_{3})$, where $A_{1}=3$, $A_{2}=5$, $A_{3}=3.2$, $f_{1}=3.7$, $f_{2}=5.1$, $f_{3}=5.2$, $\theta_{1}=11.8$, $\theta_{2}=33.5$, $\theta_{3}=63.9$. The input signal is shown in Figure 7. The sampling frequency is 100 Hz and ds is 0.1 Hz. Set different frequency ranges for regional DFT calculation. See Table 2 for the number of calculation data and calculation time results. By changing the area range [$F_{L}$, $F_{H}$], the number of operation data can be changed to greatly improve the calculation time. In the article, three intervals of DFT were calculated for the input signal, namely [0, 50], [3.5, 5.5], [5, 5.5]. The calculation results are shown in Figure 8, Figure 9 and Figure 10.

If the accuracy of the obtained $f^{\prime }$ is high, only the amplitude and phase at that frequency are calculated. Because the accuracy of the frequency domain abscissa is set to $ds=f^{\prime }$, the frequency $f^{\prime }$ corresponds to the first point in the frequency domain coordinate, and the required results are obtained by directly calculating the amplitude and phase angle of k=1. The sampling frequency is $f_{s}$, and the number of sampled data is $N=f_{s}/ds$. According to Equation (11), if only the amplitude and phase at frequency $f^{\prime }$ are taken, the formula for calculating the amplitude at the corresponding frequency is [13]:

(12) $$Y\left(k\right)=\frac{2}{N}\sum_{n=0}^{N-1}x\left(n\right)e^{-\frac{j2\pi nk}{N}},\;k=1.$$

(13) Re⁡(Y(k))=2N∑n=0N−1x(n)cos⁡(2πnk/N), k=1.

(14) Im⁡(Y(k))=2N∑n=0N−1x(n)sin⁡(2πnk/N), k=1.

The formula for calculating the phase angle is [14]:

(15) Y_Angle(k)=tan−1⁡Re⁡(Y(k))Im⁡(Y(k)), k=1.

## 5. Engineering Practice and Verification

This study takes the actual 1150 mm six-high reversible rolling mill as the experimental object to conduct engineering application research and algorithm validation for roll eccentricity detection. The parameters of the experimental rolling mill roll system are: the diameters of the upper and lower support rolls are 1018.8 mm and 1052 mm, respectively; the diameters of the upper and lower intermediate rollers are 392.2 mm and 396.36 mm, respectively; the diameters of the upper and lower work rolls are 324.25 mm and 325.72 mm, respectively. Rapid detection of roll eccentricity has been achieved in the AGC system of the rolling mill.

**Figure 11:**
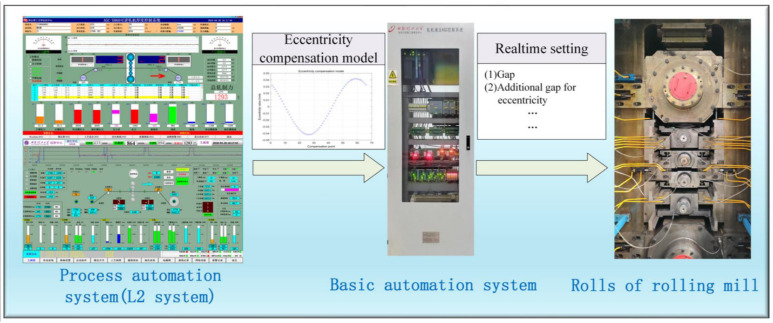
Introduces the roll and cold-rolling mill control system of a six-high reversible cold rolling mill.

This study is based on a two-level computer control system that combines a basic automation level (L1) and process automation level (L2). The L2 system uses an industrial control computer to receive and store field data and high-level language programming to analyze and calculate the roll eccentricity signal. Then establish an eccentric model and send it to the L1 control system. The L1 control system adopts a hardware platform based on Siemens S7-400PLC and Siemens FM458 to receive the eccentricity model and perform eccentricity compensation control according to the eccentricity model. The 1150 mm six-high reversible cold-rolling mill, roll, and control system are shown in Figure 11.

The data used to detect the eccentricity signal of the rolling roller come from the rolling force and outlet thickness deviation data collected on site. The specific steps include:

1. Conduct zero-pressure-dependence experiments in the roll gap control mode after each roll change.
2. Collect rolling force data when the rolling pressure is in an open loop state.
3. Generate a roll eccentricity model using the collected data.

**Figure 12:**
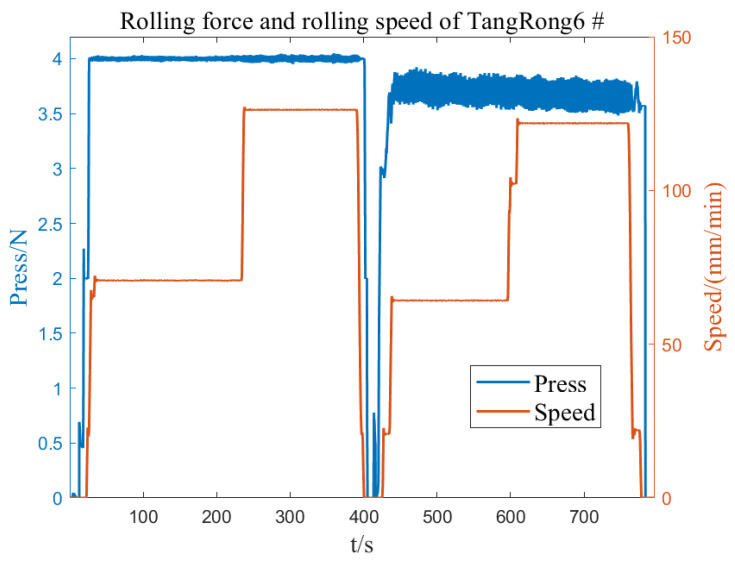
Rolling force and rolling speed of TangRong6 #.

**Figure 13:**
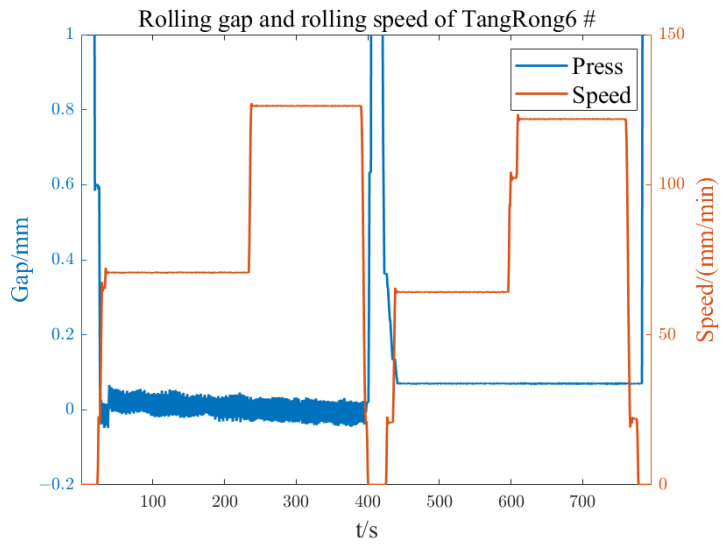
Rolling gap and rolling speed of TangRong6 #.

**Figure 14:**
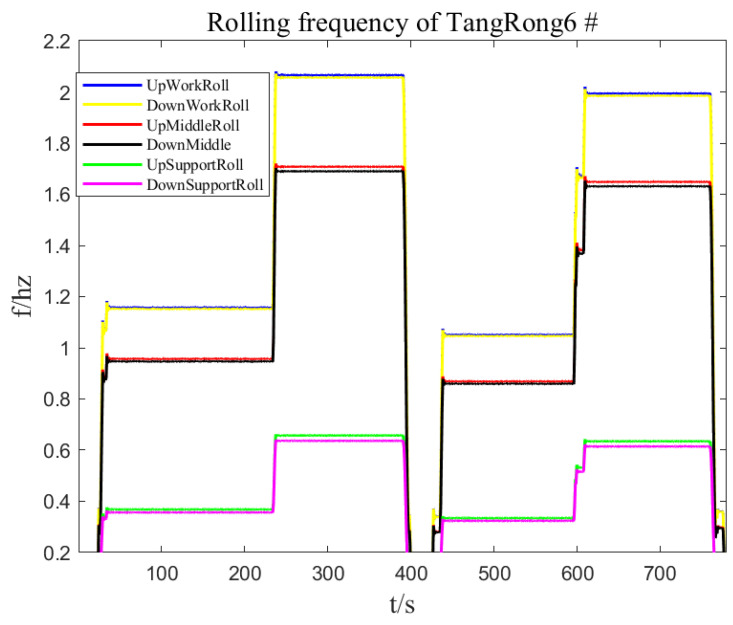
Rolling frequency of TangRong6 #.

Under the condition of not being affected by the roller piece, the rolling force can fully reflect the state of the roller and establish an accurate model for the eccentricity of the roller. During the normal rolling process, the parameters of the eccentricity model may also change due to changes in working conditions. Therefore, during the rolling process, thickness fluctuation data of the export strip steel are collected for online detection of roll eccentricity, and the amplitude and initial phase angle of each harmonic in the established zero-pressure-dependent eccentricity model are corrected. Figure 12 and Figure 13 show the rolling force and rolling speed data collected on site under zero-pressure-dependent conditions. According to the speed curve, they can be divided into four stages. The rolling force has obvious roll eccentricity in the third and fourth stages, and the roll gap is in the first and fourth stages. There is obvious eccentric disturbance in the second stage. The relationship between roller speed and strip speed is T=πD/(1000v/60), where the roller diameter D is in mm and the strip speed v is in m/min. The speeds of six rollers can be obtained as shown in Figure 14 and the rotational frequency of the rollers is the frequency of the eccentric disturbance generated. Calculate the DFT and regional DFT values corresponding to the four speed stages. Then, the L2 computer sends the detected roll eccentricity data to the L1 controller, which compensates the eccentricity model in reverse to the roll gap control circuit.

According to the rolling speed of a 6 # rolling mill, it is divided into four stages. The number of DFT operation data in each stage is 20,000, the number of regional DFT operation data is 9, the frequency domain accuracy is 0.005 Hz. DFT, and regional DFT operations are performed in each stage. The roll gap is analyzed in the first and second stages, and the rolling force is analyzed in the third and fourth stages. The calculation results are shown in Table 3 [15]. 

From the speeds of each roller in Figure 13, it can be seen that the calculated eccentricity disturbance frequency at each stage corresponds to the rotation frequency of the upper support roller, thus verifying that the eccentricity disturbance is the eccentricity of the upper support roller.

Using regional DFT for roll eccentricity detection, the detection time has decreased from over 500 s to less than 0.2 s, and the calculation time has been shortened by 3000 times. The online identification effect is good. The experimental results show that this method can quickly detect roll eccentricity disturbance and achieve online and efficient detection of roll eccentricity, which has a certain engineering application significance.

## 6. Results

To achieve rapid identification of the source and a mathematical model of roll eccentricity signals, this paper proposes a method for identifying and detecting roll eccentricity signals using sparse Fourier transform (SFT) and regional DFT. The application of this method and roll eccentricity compensation control in practical engineering was studied through experiments [1]. The innovation of this method is that it makes full use of the rapidity of SFT and the regioselectivity of DFT. On the premise that the roll diameter is known, the signal spectrum, amplitude, and phase are identified online through the regional DFT, which improves the accuracy and speed of roll eccentricity signal detection. This method does not have particularly complex calculation steps and is easy to implement through computer programs, making it suitable for engineering applications. When this method is applied in engineering experiments, it can accurately identify the source of roll disturbance, quickly update and replace the problematic rolls, and improve the online recognition efficiency by more than 3000 times. It has good results in online detection and recognition of roll eccentricity signals, greatly improving engineering application efficiency, and ultimately achieving the goal of improving the accuracy of strip outlet thickness. In the study, the limitations of this method were also found, as it is necessary to estimate the rotational frequency of each roll of the rolling mill in advance. The setting of a frequency domain accuracy also affects the accuracy of identification results and the length of operation time. Therefore, in future work, further research will be conducted on adaptive identification capabilities and improving the identification accuracy of algorithms.

## Figures and Tables

**Table 1:** Input signal SFT calculation results.

Input Signal	Signal 1	Signal 2	Signal 2
Input signal amplitude	2	3	0.3
SFT calculation results	1.9950	0.2983	2.9879
error	0.25%	0.5667%	0.4033%

**Table 2:** Regional DFT calculation results.

DFT Calculation Range	Calculate the Number of Data	Calculation Time (Seconds)	Calculation Time Proportion
[0, 50]	1000	0.466564	100%
[3.5, 5.5]	21	0.010062	2.157%
[5, 5.5]	6	0.004874	1.045%

**Table 3:** Calculation results of eccentric disturbance of 6 # rolling mill.

Roll Speed Stage	Roll Speed First Stage	Roll Speed Second Stage	Roll Speed Third Stage
DFT operation time/s	521.763166	522.359140	522.796530
Regional DFT frequency range/Hz	[0.35, 0.39]	[0.64, 0.68]	[0.32, 0.36]
Regional DFT operation time/s	0.143489	0.150094	0.141177
Regional DFT maximum/Hz	0.37 Hz	0.66 Hz	0.335 Hz

## Data Availability

The data used to support the findings of this study are available from the corresponding author upon request.

## References

[1] Yang Z., Liu D., Zheng G. (2022). Roll Eccentricity Signal Detection and Its Engineering Application Based on SFFT-IAA. Appl. Sci..

[2] Hao L., Kuan L., Okyay K., Shen Y., Mingyi H., Hao Z. (2020). A Robust Data-Driven Fault Detection Approach for Rolling Mills with Unknown Roll Eccentricity. IEEE Trans. Control. Syst. Technol..

[3] Shanfeng G., Lei X., Yongkang L., Jiwen J. (2022). Roll eccentricity extraction and compensation based on MPSO-WTD and ITD. PLoS ONE.

[4] Pengcheng Y., Hua Q., Jihong Z., Meng W. (2020). Newtonian-Type Adaptive Filtering Based on the Maximum Correntropy Criterion. Entropy.

[5] Veerendra D., Alagirisamy M. (2020). Adaptive Beamformers for High-Speed Mobile Communication. Wirel. Pers. Commun..

[6] Wenhui Z., Hao Z., Guofan J. (2021). Single-Fourier transform based full-bandwidth Fresnel diffraction. J. Opt..

[7] Xiaoyi D., Dong C., Xianwei G., Xiaohong F., Jifang J. (2020). Research and implementation of correlation power analysis based on wavelet transform. Appl. Res. Comput..

[8] Guangna Z. (2022). Research on safety simulation model and algorithm of dynamic system based on artificial neural network. Soft Comput..

[9] Giulia F., Enrico M. (2017). Steerable Discrete Fourier Transform. IEEE Signal Process. Lett..

[10] Hu J., Wang Z., Qiu Q., Xiao W., Lilja D.J. Sparse Fast Fourier Transform on GPUs and Multi-core CPUs. Proceedings of the 2012 IEEE 24th International Symposium on Computer Architecture and High Performance Computing.

[11] Feng Z. (2007). Application of atomic decomposition to gear damage detection. J. Sound Vib..

[12] Bittens S., Ruochuan Z., Iwen M.A. (2019). A deterministic sparse FFT for functions with structured Fourier sparsity. Adv. Comput. Math..

[13] Chen C., Wu J., Miao C.W.X., Bu X. On a New SNR Estimation Approach with Polar Codes. Proceedings of the 2021 IEEE 4th Advanced Information Management, Communicates, Electronic and Automation Control Conference (IMCEC).

[14] Jishy K., Lehmann F. (2013). A Bayesian track-before-detect procedure for passive radars. EURASIP J. Adv. Signal Process..

[15] Zhen Y. (2022). A New Evaluation Method for Product Service System Scheme Based on Analytic Network Process and Niche Theory. IEEE Access.

